# Supplementary material for: An Overview of SARS-CoV-2 Etiopathogenesis and Recent Developments in COVID-19 Vaccines
Source: Biomolecules. 2023 Oct 24;13(11):1565. doi: 10.3390/biom13111565 (PMC10669259; doi:10.3390/biom13111565)
Supplement: Supplementary file 1 [file biomolecules-13-01565-s001.zip › biomolecules-2657731-supplementary.pdf]

Supplementary Table S1. List of antiviral drugs recommended for emergency use against COVID-19

Antiviral drug	Dose	Route	Manufacturer	Indications for use	Trials/Studies involved	Side effects
PAXLOVID (Nirmatrelvir and Ritonavir tablets co-packaged	Nirmatrelvir - 300mg or 150mg Ritonavir-150mg as prescribed	Oral	Pfizer labs, USA	EUA for the treatment of mild-to-moderate COVID-19 in adults and children 12 years of age and older weighing at least 88 pounds (40 kg) who are at high risk for progression to severe COVID-19, including hospitalization or death.	EPIC-HR trial	Altered sense of taste and Diarrhoea
LAGEVRIO (Molnupiravir capsules)	800 mg	Oral	Merck Sharp & Dohme LLC, USA	EUA for the treatment of adults at high risk for progression to severe COVID-19, including hospitalization or death or whom alternative COVID-19 treatment options approved or authorized by FDA	MOVe-OUT trial	Diarrhoea, nausea and dizziness
VEKLURY Remdesivir	200mg first dose and 100mg subsequent doses	IV infusion	Gilead Sciences, Inc., USA	EUA for the treatment of adults and children at high risk and hospitalized with severe disease	ACTT-1; CATCO; DisCoVeRy; WHO Solidarity Trial	Increased liver transaminases

The table summarizes various antiviral drugs used alone or co-administered with another, their dosage, route of administration, manufacturer, indications for use, clinical trials and side effects [100,102]. IV-intravenous; EUA-Emergency Use Authorization.

Supplementary Table S2. List of monoclonal antibodies recommended for emergency therapeutic use against COVID-19

Antibody	Dose and Route	Route	Manufacturer	Indications for use	Half-life (days)	Variants	Trial name /Studies involved	Adverse events
Bebtelovimab	175 mg	IV injection	Eli Lilly and Company, USA	EUA for high-risk mild-to-moderate COVID-19 in adults and pediatric patients (12 years of age and older weighing at least 40 kg):	11.5	All VOC and VOI	Blaze-4 trial	None except infusion-related reactions, pruritus and rash
Bamlanivimab and Etesevimab coadministration	700 mg and 1400 mg respectively	IV infusion	Eli Lilly and Company, USA	EUA for high-risk mild-to-moderate COVID-19 in adults and pediatric patients (12 years including neonates	20.9 and 32.6	All VOC and VOI	Blaze 1 and 2 trials	Nausea, dizziness, pruritus, anaphylaxis
ACTEMRA (tocilizumab	8mg/kg or 12 mg/kg body weight for patients at >30kg and ≤30 kg body weight respectively	IV infusion	Genentech, Inc, USA	EUA for COVID-19 in hospitalized pediatric patients (2 years of age and older) receiving systemic corticosteroids and requiring supplemental oxygen, non-invasive or invasive mechanical ventilation, or extracorporeal membrane oxygenation (AVOID use of any live vaccine)	13	All VOC and VOI	Recovery trial EMPACTA, COVACTA, REMDACTA studies	Constipation, anxiety, diarrhea, insomnia, hypertension and nausea
EVUSHELD (tixagevimab and cilgavimab coadministration)	300 mg each	IM injection	Astrazeneca, USA	EUA for COVID-19 pre-exposure prophylaxis of COVID-19 in adults and pediatric individuals (12 years of age and older weighing at least 40 kg):	87.9 and 82.9	All VOC and VOI	PROVENT and STORM CHASER trials	Headache, fatigue, and cough.
REGEN-COV (casirivimab and imdevimab coadministration)	600 mg each	IV infusion or SC injection	Regeneron Pharmaceuticals, Inc., USA	EUA for high-risk COVID-in adults and pediatric patients (12 years of age and older weighing at least 40 kg)	31.8 and 26.9	Alpha, Beta, Gamma, Delta and Kappa	COV-2067, COV-2069 and COV-2093	Dizziness, nausea, rash, chills, and lymphadenopathy
SOTROVIMAB	500 mg	IV infusion	Glaxo Smith Kline LLC, USA	EUA for high-risk COVID-in adults and pediatric patients (12 years of age and older weighing at least 40 kg)	49	All VOC and VOI	COMET-ICE and COMET-TAIL trial	Rash, diarrhoea, infusion-related reactions, and hypersensitivity

The table summarizes various monoclonal antibodies administered alone or co-administered with another, their dosage, route of administration, manufacturer, indications for use, half-life, clinical trials and adverse events [103]. IV-intravenous; IM-intramuscular; SC-subcutaneous; mg-milligram; EUA-Emergency Use Authorization; VOC-Varients of Concern; VOI- Variants of Interest.

Supplementary Table S3. Description of various COVID-19 vaccine types currently in clinical trials							
**S.No**	**Vaccine type**	**Developer**	**Name**	**Approved countries**	**Phase**	**Route**	**Dose**
1	Protein subunit	Anhui Zhifei Longcom	Zifivax	4	3	IM	Day 0 + 28 + 56
2	Protein subunit	Bagheiat-allah University of Medical Sciences	Noora vaccine	1	3	IM	Day 0 + 21 + 35
3	Inactivated Virus	Bharat Biotech	Covaxin	14	3	IM	Day 0 + 14
4	NRVV	Bharat Biotech	iNCOVACC	1	3	IN	Day 0
5	Protein subunit	Biological E Limited	Corbevax	2	3	IM	Day 0 + 28
6	NRVV	CanSino	Convidecia	10	3	IM	Day 0 + 21
7	NRVV	CanSino	Convidecia Air	2	4	IH	Day 0
8	Protein subunit	Center for Genetic Engineering and Biotechnology (CIGB)	Abdala	6	3	IM	Day 0 +28 + 56
9	Inactivated Virus	Chumakov Center	KoviVac	3	3	IM	Day 0 + 14
10	NRVV	Gamaleya	Gam-COVID-Vac	1	3	IM	Day 0 + 21
11	NRVV	Gamaleya	Sputnik Light	26	3	IM	Day 0
12	NRVV	Gamaleya	Sputnik V	74	3	IM	Day 0 + 21
13	RNA	Gennova Biopharmaceuticals Limited	GEMCOVAC-19	1	2/3	IM	Day 0 + 28
14	Inactivated Virus	Health Institutes of Turkey	Turkovac	1	3	IM	Day 0 + 21
15	Protein subunit	Instituto Finlay de Vacunas Cuba	Soberana 02	4	3	IM	Day 0 + 28
16	Protein subunit	Instituto Finlay de Vacunas Cuba	Soberana Plus	2	3	IM	Booster for Soberana 02
17	NRVV	Janssen (Johnson & Johnson)	Jcovden	113	4	IM	Day 0 or Day 0 +56
18	Protein subunit	Livzon Mabpharm Inc	V-01	1	3	IM	Day 0 + 21
19	VLP	Medicago	Covifenz	1	3	IM	Day 0 + 21
20	Protein subunit	Medigen	MVC-COV1901	4	4	IM	Day 0 + 28
21	RNA	Moderna	Spikevax	88	4	IM	Day 0 + 28
22	RNA	Moderna	Spikevax Bivalent Original/Omicron BA.1	38	3	IM	Day 0 + 55
23	RNA	Moderna	Spikevax Bivalent Original/Omicron BA.4/BA.5	33	3	IM	Day 0 + 55
24	Protein subunit	National Vaccine and Serum Institute	Recombinant SARS-CoV-2 Vaccine (CHO Cell)	1	3	IM	Day 0
25	Protein subunit	Novavax	Nuvaxovid	40	3	IM	Day 0 + 21
26	Inactivated Virus	Organization of Defensive Innovation and Research	FAKHRAVAC (MIVAC)	1	1	IM	Day 0 + 14
27	NRVV	Oxford/AstraZeneca	Vaxzevria	149	3	IM	Day 0 + 56
28	RNA	Pfizer/BioNTech	Comirnaty	149	4	IM	Day 0 + 21
29	RNA	Pfizer/BioNTech	Comirnaty Bivalent Original/Omicron BA.1	35	3	IM	Day 0 + 21
30	RNA	Pfizer/BioNTech	Comirnaty Bivalent Original/Omicron BA.4/BA.5	33	3	IM	Day 0 + 21

31	Protein subunit	PT Bio Farma	IndoVac	1	3	IM	Day 0 + 28
32	Protein subunit	Razi Vaccine and Serum Research Institute	Razi Cov Pars	1	3	IM	Day 0 + 21 +51
33	Inactivated Virus	Research Institute for Biological Safety Problems (RIBSP)	QazVac	2	3	IM	Day 0 + 21
34	Protein subunit	Sanofi/GSK	VidPrevtyn Beta	30	3	IM	Day 0 + 21
35	NRVV	Serum Institute of India	Covishield (Oxford/ AstraZeneca formulation)	49	3	IM	Day 0 + 84
36	Protein subunit	Serum Institute of India	COVOVAX (Novavax formulation)	6	3	IM	Day 0 + 21
37	Inactivated Virus	Shenzhen Kangtai Biological Products Co	KCONVAC	2	3	IM	Day 0 + 28
38	Inactivated Virus	Shifa Pharmed Industrial Co	COVIran Barekat	1	2/3	IM	Day 0 + 14
39	Inactivated Virus	Sinopharm (Beijing)	Covilo	93	4	IM	Day 0 + 21
40	Inactivated Virus	Sinopharm (Wuhan)	Inactivated (Vero Cells)	2	4	IM	Day 0 + 21
41	Inactivated Virus	Sinovac	CoronaVac	56	4	IM	Day 0 + 14
42	Protein subunit	SK Bioscience Co Ltd	SKYCovione	1	3	IM	Day 0 + 28
43	Protein subunit	Takeda	TAK-019 (Novavax formulation)	1	3	IM	Day 0 + 14 + 28
44	RNA	Takeda	TAK-919 (Moderna formulation)	1	3	IM	Day 0 + 28
45	Inactivated Virus	Valneva	VLA2001	33	3	IM	Day 0 + 21
46	Protein subunit	Vaxine/CinnaGen Co.	SpikoGen	1	3	IM	Day 0 + 21
47	Protein subunit	Vector State Research Center of Virology and Biotechnology	Aurora-CoV	1	3	IM	Day 0 + 21
48	Protein subunit	Vector State Research Center of Virology and Biotechnology	EpiVacCorona	4	3	IM	Day 0 + 21
49	RNA	Walvax	Awcorna	1	3	IM	Day 0 + 14 or Day 0 + 28
50	DNA	Zydus Cadila	ZyCoV-D	1	3	ID	Day 0 + 28 + 56
**Vaccines Granted Emergency Use Listing (EUL) by WHO**							
1	Protein subunit	Serum Institute of India	COVOVAX (Novavax formulation)	6	3	IM	
2	Protein subunit	Novavax	Nuvaxovid	40	3	IM	Day 0 + 21
3	RNA	Moderna	Spikevax	88	4	IM	Day 0 + 28
4	RNA	Pfizer/BioNTech	Comirnaty	149	4	IM	Day 0 + 21
5	NRVV	CanSino	Convidecia	10	3	IM	Day 0 + 21
6	NRVV	Janssen (Johnson & Johnson)	Jcovden	113	4	IM	Day 0 or Day 0 +56
7	NRVV	Oxford/AstraZeneca	Vaxzevria	149	3	IM	Day 0 + 56
8	NRVV	Serum Institute of India	Covishield (Oxford/ AstraZeneca formulation)	49	3	IM	Day 0 + 84
9	Inactivated Virus	Bharat Biotech	Covaxin	14	3	IM	Day 0 + 14
10	Inactivated Virus	Sinopharm (Beijing)	Covilo	93	4	IM	Day 0 + 21
11	Inactivated Virus	Sinovac	CoronaVac	56	4	IM	Day 0 + 14

The table summarizes the various types of COVID-19 vaccines along with list of vaccines granted Emergency Use Listing (EUL) by WHO, developer, vaccine name, countries approved for use, phase, route of administration and number of doses with days [51,100]
